# Community empowerment through participation in a tsetse control project in the Democratic Republic of Congo

**DOI:** 10.1371/journal.pgph.0001325

**Published:** 2023-06-14

**Authors:** Catiane Vander Kelen, Alain Mpanya, Epco Hasker, Erick Miaka, Ruth Nzuzi, Justin Pulford, Steve Torr, Dennis Perez Chacon

**Affiliations:** 1 Public Health, Institute of Tropical Medicine, Antwerp, Belgium; 2 Coordination, Programme National de Lutte contre la Trypanosomiase Humaine Africaine, Kinshasa, Democratic Republic of Congo; 3 International Public Health, Liverpool School of Tropical Medicine, Liverpool, United Kingdom; 4 Vector Biology, Liverpool School of Tropical Medicine, Liverpool, United Kingdom; 5 Epidemiology, Pedro Kouri Institute of Tropical Medicine Center for Diagnostic and Reference Research, La Habana, Cuba; University of Washington, UNITED STATES

## Abstract

Gambiense Human African Trypanosomiasis (g-HAT) is a neglected tropical disease caused by trypanosomes transmitted by tsetse flies. In 2017, a pilot community-based project was launched in three villages in DRC with the overall goal of empowering community members to control tsetse using Tiny Targets which attract and kill tsetse. In this paper, we assess the community participation process in these three pilot villages over >4 years and evaluate to what extent this resulted in the empowerment of communities. We conducted a qualitative study using a participatory research approach. Together with community members of the three pilot villages from the endemic Kwilu province, we evaluated changes in project participation, community empowerment and perception of future participation at three different time points (September 2017, September 2018 and November 2021) over a 4-year period using participatory workshops and focus group discussions (FGD). We used a thematic content approach to analyse both workshop notes and FGD transcripts. The community identified five indicators to evaluate participation: (1) Leadership & Ownership, (2) Organisation & Planning, (3) Willingness, (4) Autonomy and (5) Community Involvement. The participation experience described by community members was characterised by a rapid growth of empowerment in the first year and sustained high levels thereafter. Community participants were willing to engage in potential future projects and continue to be supported by their Tiny Target project partner. However, they identified an imbalance in the power relationship within the committee and with the Tiny Target partners that limit the extent of empowerment attained. The intervention had broader benefits of community empowerment but this was limited by perceptions of being part of wider “top down” programme and by stakeholders attitude toward community participation. If empowerment is to be an important objective of projects and programmes then the needs identified by communities must be recognised and attitude of sharing power encouraged.

## Introduction

Human African Trypanosomiasis (HAT) or sleeping sickness is a neglected tropical disease (NTD) afflicting poor and rural populations in sub-Saharan Africa (SSA) [1]. HAT is caused by subspecies of *Trypanosoma brucei* transmitted by tsetse flies (*Glossina*) [2]. There are two forms of HAT: an anthroponotic form known as gambiense HAT (g-HAT) and a zoonotic form known as rhodesiense HAT (r-HAT) [3]. g-HAT accounted for 85% of all global cases reported in 2021 (747/802) of which 70% (425/747) were reported in Democratic Republic of Congo (DRC) [4]. If left untreated, HAT is almost always fatal [5].

In 2012, the World Health Organization (WHO) presented a plan to eliminate g-HAT, first by reducing its incidence to very low levels (<1 new case/year/10,000 people in 90% foci and <2000 cases/year reported globally) and, in a second stage, by interrupting transmission completely by 2030 [6, 7]. Active case detection and case treatment are the mainstays of efforts against g-HAT [4], supported by tsetse control in settings where elimination may not be achieved rapidly [3, 8–12].

Tiny Targets, small (50 x 25 cm) panels of cloth and mesh impregnated with insecticide, created specifically to control tsetse provide a cost-effective method of controlling the vector of HAT [3, 13–16]. This simple and cheap tool can be implemented by communities living in HAT affected areas [17].

Since the Alma Ata Declaration in 1978, community participation has been advocated as an essential factor towards the achievement of health care goals [18]. However, rigorous analyses of community-based projects face many challenges. These include: i) the absence of a widely-accepted definition of “community” and “participation”, (ii) the assumption that involving communities automatically fosters community interest, empowerment and self-sustainability [19], (iii) the lack of evidence that community participation contributes to programme outcomes, (iv) donor disinterest because of this lack of evidence, (v) neglecting the consideration of factors related to power and control as a key to empower people [20].

In the 1980s and 1990s, several tsetse control interventions involving the deployment of insecticide-treated targets by local communities were implemented in many countries [21–25]. While the interventions were cost-effective, the majority stopped once donors withdrew, even when human and/or animal trypanosomiasis was still a burden [26, 27]. Community participation was often seen by programme planners and donors as a way of transferring the labour and material costs of tsetse control to communities [28, 29]. Consequently, analyses of these community-based interventions focused more on economic costs and effectiveness while largely ignoring the context and social process of participation. Since then, studies about tsetse control and community participation are exceedingly scarce with only one community-based study identified, this being an investigation of tsetse control in three villages in Uganda [30].

Community participation is approached in different ways by planners and programmes. According to Rifkin, community participation approaches have ranged from ‘top down’ to ‘bottom up’. Top down is when community participation is considered a means to the end of attaining health improvement objectives and where participation is primarily in the form of community members doing what health professionals tell them. Bottom up is when community participation is seen as an end in itself and where community members assume leadership roles supported by health professionals [31–34].

We position community participation within an empowerment approach as defined by Rifkin in 1988: ‘a social process whereby specific groups with shared needs living in a defined geographic area actively pursue identification of their needs, take decisions and establish mechanisms to meet those needs.” [32, 34].

In 2017, a pilot community-based project was launched in three villages in DRC with the overall goal of empowering people to control tsetse using Tiny Targets. Initially, we assessed the operational feasibility of the project and evaluated whether this approach could form part of the national strategy for HAT elimination [17]. Following the success of the community-based project, the approach was scaled up to include 96 villages. In the present paper, we assess the community participation process of the three initial pilot villages over >4 years to evaluate to what extent this resulted in empowering communities.

## Material and methods

### Study area and population

**Fig 1:**
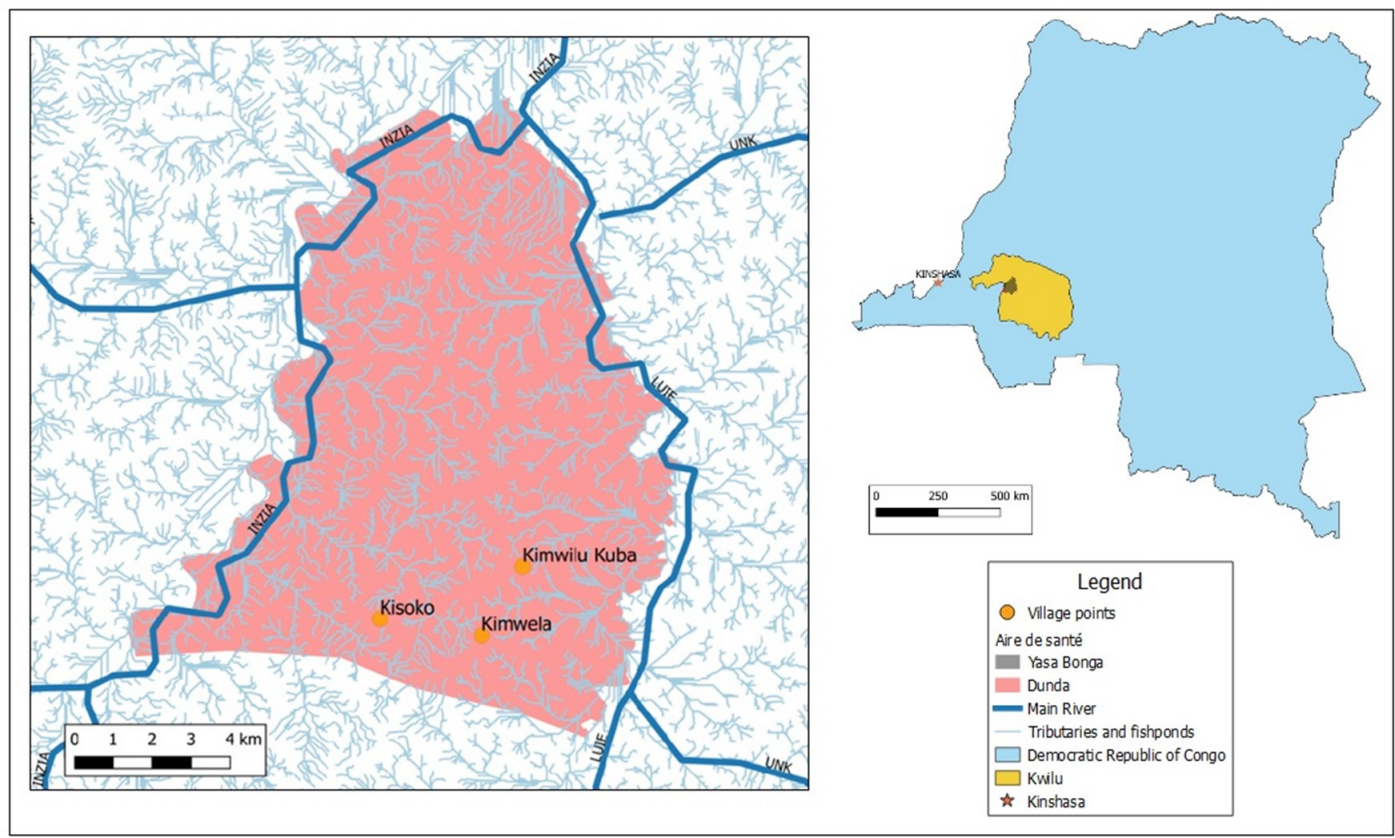
Location of the three pilot villages in the Dunda health area of Yassa Bonga health zone in Kwilu province, and the rivers, tributaries and fishponds. DRC and Provinces source is GADM (https://gadm.org/license.html); Health Zone and Health Areas (https://data.humdata.org/dataset/drc-health-data); (Rivers) https://data.humdata.org/dataset/hydrographie-lineaire-rdc-drc-water-courses (Credit: ITM and LSTM).

The project was launched in three pilot villages from the Dunda health area in the south west of the Yasa Bonga health district (see Fig 1): Kimwilu Kuba (Pop: 1441; Cases: 4), Kimwela (Pop: 1200; Cases: 3) and Kisoko (Pop: 1005. Cases: 3).

### Project implementation

Following the demonstration that the community-based approach to Tiny Target deployment and management was feasible and effective, the strategy was included in the PNLTHA tsetse control programme and was scaled up to 96 villages in 2019. Three changes were made in the scale-up for the project to be manageable. First, responsibility for guidance, support and material distribution was assumed by four PNLTHA agents, replacing the research team. Second, an incentive of 5 United State Dollars (USD) for each deployment was introduced so as to be in line with payments made for other sleeping sickness activities. Third, Tiny Targets were delivered assembled and the number was limited to 150–200 targets per deployment to reduce workload and improve distribution and deployment. Previously, local communities assembled targets themselves and were provided with, effectively, an unlimited supply.

### Framework and procedures to evaluate empowerment

Our empowerment evaluation was guided by a framework proposed by Rifkin et al. in 1988 [32] and adapted by Draper et al. in 2010 [33]. This framework is a continuum that goes from mobilisation (community does what the professional advises), to collaboration (community contributes with time, materials and/or money), to empowerment (community plans and manages health activities with professional support) [32]. To evaluate participation, Rifkin et al. proposed five indicators: 1) needs assessment evaluation (implication in community needs identification); 2) sense of leadership (scope of community interests representation); 3) programme organisation involvement (involvement of existing community structures); 4) programme management level (community autonomy to implement activities); and 5) resource mobilization contribution (community resources invest in the project). Then different hypothetical situation are described regarding mobilisation, collaboration and empowerment for each indicators. The results are generally presented in a spidergram for more clarity [32, 33]. Draper et al. proposed a adapted version where indicators can be different and chose according to context preferably by or with the community. In our study we will use the adapted version.

We conducted a three-step evaluation with each of the vector control committees between March 2018 and November 2020.

*The first step* consisted of participatory workshops held with each of the three vector control committees in March 2018. The objective was to discuss how committee members defined community participation and, together with the research team, agree upon a defined set of community participation indicators with the purpose of evaluating them repeatedly over time.

*The second step* took place in September 2018 and another set of participatory workshops were organised with committee members. A baseline evaluation and a post-implementation evaluation were done where scores on each indicator were compared between the first Tiny Target deployment (August 2017) and the situation in September 2018, where three different deployment had been realised.

*The third step* consisted of another set of participatory workshops in November 2020, after the seventh Tiny Target deployment and more than a year after the operational changes introduced by the scale up of the community-based activity, to re-evaluate indicators and any perceived changes in assigned score since the last evaluation.

**Fig 2:**
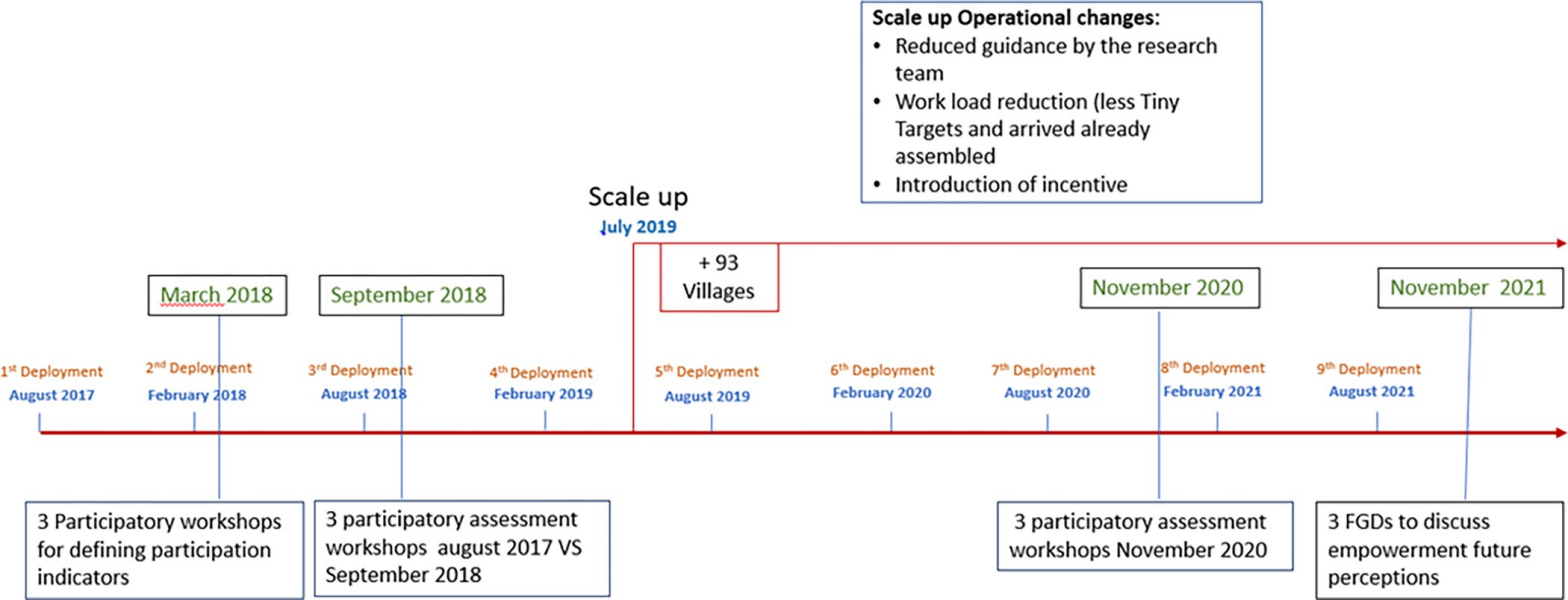
Data collection and project operational evolution timeline.

In November 2021, we decided to carry out additional Focus Group Discussions (FGDs) to explore how committee members perceived their future participation and empowerment until sleeping sickness eradication is achieved and beyond. Fig 2 summarises the evaluation timeline.

A total of nine participatory workshops and three FGDs were conducted. All were held with the vector control committee members of the three pilot villages (see Table 1 for committee characteristics) separately in a classroom outside school hours. They were conducted in Kikongo by a Kikongo-speaking anthropologist (RN), assisted by a local assistant and the main researcher (CVK). Each workshop lasted around two hours using participatory learning tools, such as tables and spidergrams depicted on a big flipchart as a visual tool to aid discussion. FGDs lasted around one hour each using a topic guide. Workshops and FGDs were audio-recorded with permission of the participants, and notes were taken by the local assistant and the main researcher (CVK).

**Table 1:** Vector committee characteristics.

Village	Female	Male	Total
Kimwilu Kuba	2	10	12
Kimwela	5	12	17
Kisoko	5	11	16
TOTAL	12	33	45

### Data analysis

The assignment of participation scores was done with each committee in their respective village. The research team then compiled the indicators and evaluation results for further analysis of changes in participation over time. Audio-recorded FGDs and workshops were translated from Kikongo to French and transcribed by external translators. Field notes were systematically recorded in a journal after each workshop and FGD. All transcripts and field notes were cross-checked by the research team to ensure accuracy. CVK and RN analysed the data using a thematic content analysis approach [35]. This method combines a deductive approach, through predefined themes in the FGDs questions, meetings or observational guides, and an inductive approach for which themes were identified through careful reading and re-reading of the data. If patterns were recognized, these emerging themes became the categories for analysis. NVivo software (version 11; QSR International, Melbourne, Australia) was used to conduct the data analysis.

### Ethical approval

The research protocol was reviewed and approved by the ethical committees of the Institute of Tropical Medicine in Antwerp, Belgium (ref: 1157/17) and the public health school of the University of Kinshasa (ref: ESP/CE/029/2017). Prior to data collection the authorities of Yasa Bonga health district and village chiefs were informed about the objective of the research and gave permission to conduct the study. All participants were informed about the objective of the research, their voluntary participation and their right to withdraw from the study. Oral consent was audio recorded as some participants could not write and read.

## Results

Our results are presented in three subsections. The first section describes the definition and operationalisation of the indicators for the community empowerment evaluation. The second presents the empowerment process evaluation results and the third the perceived future of this empowerment until the end of the project and beyond.

### 1. Definition and operationalisation of the indicators for the evaluation of participation

All three committees defined three similar indicators: (i) ‘*Ownership & leadership’* (ii) ‘*Planning & Organisation’* and (iii) ‘*Volunteering’*. A further indicator was identified by two committees: (iv) ‘*Autonomy’*. A fifth indicator, ‘*community involvement*’, was proposed by the lead author (CVK). Indicators and their definitions were then compiled into a table by the research team alongside three hypothetical situations referring to the three stages of the participation continuum as proposed by Rifkin: mobilisation (1), collaboration (2) and empowerment (3) for each indicator [32].

The table was presented back to each committee for refinement and agreement. The research team was not sure if ‘*Leadership & ownership’* were considered as one concept or two. Committee members reaffirmed it was one concept because they defined it as “who is chief of the project” which in their view automatically implied ownership. Members agreed with all indicator definitions compiled in the table except for “*volunteering*” in which it became apparent that the research team had misunderstood the three committees’ point of view. The research team understood this indicator as “working without compensation.” However, committee members understood this as “working with willingness” and working without compensation was evidence of this willingness but not a requirement. In Kikongo language, volunteering and willingness are translated by the same word “luzolo” which explained the misunderstanding between the research team and the committees. Based on these discussions, this indicator was translated into English for this paper as ‘*willingness*’.

**Fig 3:**
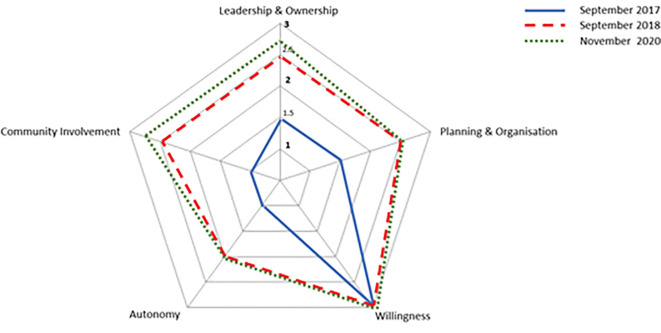
Changes in the process of community participation in the three pilot communities combined.

After discussion, a final table was consolidated to be used as a model for the evaluation (see Table 2). Committees and the research team proceeded to evaluate changes in participation at three different time points. They scored all indicators from 1 to 3 referring to the three different points on the participation continuum which goes from mobilisation to empowerment. Committee members sometimes deliberated between two hypothetical situations, so intermediate scores (i.e., 1.5, 2.5) were added to the evaluation scale. These analyses were presented as a spidergram. Fig 3 shows changes in participation over time for all three committees combined, as the committee-specific results were largely similar.

**Table 2:** Analysis grid compiling the 5 participation criteria defined by the committee members and the three points on the community participation continuum: Mobilisation (1) collaboration (2) empowerment (3).

	1 Mobilisation	2 Collaboration	3 Empowerment
**Leadership & Ownership** *« Who is the chief of the project” Or “who is taking decisions”*	The project is led by the PNLTHA and the committee support the project. The PNLTHA is perceived to be the project owner	The project is led for some activities by the committee and for other by the PNLTHA. Ownership is shared.	The project is led by the committee and PNLTHA support it. The committee feel they own the project.
**Planning and organisation** “*Who is implementing activities”*	Activities are planned and organised by the PNLTHA and decide what the committee has to do and when.	The committee plans, organises and manages some pre-determined activities under the supervision of the PNLTHA.	Activities are planned and organised by the committee who decide how and when to implement activities. The PNLTHA gives technical support
**Willingness** *“How community is contributing with its willingness*	Committee members are recruited by the PNLTHA based on their competencies and paid a salary. Money and self-interests are the main motivating factors.	Participants are designated by the community but with criteria defined by the PNLTHA. Motivation is still mainly individual but with some communal objectives.	Committee members are selected based on the project’s objectives. An individual’s motivation is related mainly to improving community well-being.
**Autonomy** *“How the project can be self-sustained by the community”*	Continuation and duration of the project depends on the PNLTHA only.	Continuation depends on the competencies acquired by the committee and external funds.	Community can sustain the project without external support.
**Community Involvement** *“How project responds to the entire community needs”*	The community diversity and the specific needs of each group are not taken into account specifically.	The interest of different groups defined by the PNLTHA are taken into account	The interests of each group are taken into account. Diversity is considered by the committee and project objectives benefit everybody.

### 2. Evaluation of the participation process: Empowerment is strengthening

From August 2017, the baseline evaluation, to September 2018, we observed a clear strengthening of community participation across four of the indicators identified by the committees; the ‘*willingness’* indicator did not strengthen as it was already considered to be at its maximum from the outset. By November 2020, the situation was maintained with a slight strengthening on two indicators. We now discuss perceived changes on each indicator in turn.

#### ‘Leadership & ownership’

Committees explained ‘*leadership and ownership’* as something to acquire. Committee members underlined the fact that as the project initiative came from outside the community, the ‘*leadership & ownership’* could not be there from the beginning. ‘*Leadership & ownership’* grew through the acquisition of knowledge via the various trainings given by the research team at the beginning of the project. Based on knowledge they already had about their environment and the new knowledge acquired they could then start to take initiatives, take decisions, lead activities and owned the project.

“*One said that they needed first to acquire the skills to be independent*. *But today they manage the committee organisation by their own*, *they manage the group members*, *they decide the rules and solve the problems*. *They are their own chief*, *not like with other projects*. *(FGD notes*, *September 2018*, *Kisoko)*

Then in the second evaluation, scores grew slightly because members in all three committees described that the hierarchy within the group disappeared over time increasing the sensation of shared ‘*Leadership & ownership’* amongst the group. ‘*Leadership & ownership’* was described, at the beginning, as held by the village chiefs -who were all part of the committees- and then increasingly shared over time.

*“The chief decided a lot, because it is often like that with projects. It is the NGO who comes and discusses with the chief, but here we understand everybody had to participate and take responsibility and finally the hierarchy had disappeared.” (FGD, November 2020, Kisoko)*

Whilst committee members did not want to reduce the score for this indicator, they described some limits to it. With the operational changes, they reported that the relationship with the external partner changed and they sensed a loss of control regarding the project. For instance, committee members complained that some aspects of the Tiny Target programme were withdrawn from their control such as assembling the Tiny Targets or the cessation of reserve stock provision which meant that they were no longer able to replace damaged Tiny Targets. As the research team stepped back, the PNLTHA, who took the supporting role, was perceived as an active actor with the power of ending the community-based project.

*“We are motivated Maman! But the project depends on the PNLTHA. If we don’t have the material there is no way for this project to continue. We need the PNLTHA they don’t have to abandon us! Last time the team just came to give the Tiny Targets but the rest of the material was missing. We try to mention this to them, but nothing. At the end it is they who decide” (FGD, November 2020, Kimwela)*

Although they described losing control of certain aspects of the programme, they stressed how the relationship with the external partner (i.e. the PNLTHA) was essential for maintaining motivation and their ‘*willingness’*.

*“It is not only the “soap” (incentive) that motivates us. When you don’t come for long periods we are a bit discouraged, we fear you never come back. Your presence motivates us. We are congratulated even by the screening team for our work. All this motivates us to continue.” (FGD, November 2020, Kimwilu Kuba)*

#### ‘Planning and organisation’

Committee members explained how they used their own initiative such as devising a Tiny Targets maintenance system or the election of a committee president or how they planned activities to fit in their spare time.

*« One said that during the last deployment the research team did not accompany them, they gave the Tiny Targets and that was all. It was the committee who decided how to organise themselves and when to deploy.” (FGD notes, September 2018, Kimwilu Kuba)*

#### ‘Autonomy’

Committee members described how the acquisition of new skills and all initiatives they took to organise the project over time increased their autonomy. However, they were conscious of the limits to their autonomy in the sense that they relied on partners to provide the Tiny Targets and for financial support to maintain the programme. This is why according to them this indicator score could not increase further.

*“One said even if they are autonomous to manage the project through knowledge and practice acquired, they cannot make the Tiny Targets, they will never be totally autonomous financially and we have the PNLTHA as external partner to provide us material”(FGD Notes, September 2018, Kimwela)*

*‘Willingness’*, was described as the motivation and commitment of committee members to work on the project. Project success was described as inseparable from the committee’s willingness to participate. This aspect was central and described as the driving force of the participation process by committee members. This indicator was considered extremely important among participants and they scored it at the maximum.

*“One said they bring force to the project with their knowledges and willingness, without willingness from their part this project could never have worked.”(Workshop notes, September 2018, Kimwilu Kuba)*

#### ‘Community involvement’

Committee members said at the beginning they primarily concentrated on the committee constitution and activities progression. After a while, and due to the contact with the research team who emphasized this matter, they increased efforts to make the project more inclusive by ensuring everybody in the community was informed, all zones were covered and everyone respected the Tiny Targets.

At the second evaluation they scored community involvement slightly higher because in addition to the community getting more involved, the committee were also engaging with the community in new and varied ways. For instance, committee members explained that they had transmitted some technical knowledge and practices about Tiny Targets to the community. One committee had also created a communal ‘pot’ with a proportion of their financial incentive in case somebody in the community needed help with health matters.

*“In our committee we even created the “ristourne”, each time we received money from the PNLTHA we put 100 francs in a common pot, for helping us because we never know when the disease comes or other life difficulties, this money is also for the other, who are not part of the committee in case of problem” (FGD, November 2020, Kimwilu Kuba)*

### 3. Future community participation through partnership

In this section we explored the committees’ perception of the future regarding their empowerment until the end of the project and beyond.

When the project future was discussed, two committees understood Tiny Target deployment will stop when the disease has been eliminated and one expressed surprise and did not seem to have recognized that Tiny Targets would be maintained only as long as cases of g-HAT continued. Nevertheless, all three had the belief that the disease will not be eliminated by 2030 and warned that if the project stopped too soon sleeping sickness would return to previous levels. Some were also confused about the fact that the disease might be eliminated while tsetse continued to be present. The observation that tsetse were still present everywhere suggested that Tiny Targets deployment must continue.

*«The project cannot finish soon, there are still a lot of flies in our forest and many villages do not use Tiny Targets. People are travelling and can bring Sleeping Sickness to us, we are living close to the main road here. Believe me this project needs to continue, our children will find this project working still!” (FGD, November 2021, Kisoko)*

They all reiterated very clearly that the project, in its current state, needs the partnership of the PNLTHA and could not survive if PNLTHA left. They said the project was initiated by the PNLTHA and can be stopped by them at any point. They also insisted that they needed an expert to support them and bring the Tiny Targets, but also that this partnership was a real driving force for keeping their motivation and willingness to participate up, which was considered as essential as the expertise of the PNLTHA to maintaining the project.

*“At the beginning we said that the project was our project, but we still need you to make it work, the objective is to finish with sleeping sickness and you have to accompany us, you cannot abandon us like other organisations. We like working together this motivates us very much » (FGD, November 2021, Kimwilu Kuba)*

Participants emphasized that they were very keen to be involved in other projects. They stressed the skills and knowledge they acquired and the functional group that they created, the “family” relations they have built. They felt that these can be used for other activities brought by the PNLTHA or another partner. If the project must end, then they expressed their wish of being properly and officially acknowledged and not “abandoned” like previous projects were perceived to have done.

*« We have knowledge now. We have learned many things. There are other disease in our villages, if you bring us another project we will continue working the same way for other community projects. (FGD, November 2021, Kimwela)*

*“The end of the project does not mean the end of our relationship. The end depends on the PNLTHA but we like the PNLTHA to bring another project, we can work on malaria or to build drinking water system. Also if you decide it is the end we would like to have some acknowledgement like a ceremony and a plaque” (FGD, November 2021, Kisoko)*

This suggests that, even after several years, participants couldn’t see an independent role for themselves in a broader context. It also indicates that this possibility was probably not explored with the partner; they were empowered to be good partners for this project rather than something that goes beyond the program.

## Discussion

Our study provides evidence that community participation in a tsetse control project involving the use of Tiny Targets can be a source of empowerment. Community members belonging to village committees tasked with managing the deployment of Tiny Targets in their local area gained empowerment through programme ‘leadership & ownership’ (decision making and project appropriation) and ‘willingness’ (motivation to contribute to community well-being). However, the committees also identified barriers to further empowerment related to power structures and attitudes.

Empowerment was constrained by the top-down relationship with the external partner and power structures within the committee. These constraints were not immutable, with evidence of shifting power relations over the course of the study. For example, in the first phase of the evaluation, the research team was initially perceived as holding more power because they initiated the project yet committee members described rapidly gaining an increased sense of leadership and ownership which led to greater participation. Similarly, within the committees the leadership was initially held by the village chiefs. However, during the second phase of evaluation the committee members reported that power was shared more widely among committee members. These internal shifts in power have not always been reported. A study in Tanzania [36] which also employed Rifkin’s community participation framework found leadership was regarded as being restricted to the village leader who made almost all decisions without consulting village councils. The community recognized this, but found it totally acceptable as it reflected the expected power structure.

Which is not to suggest that traditional power structures were fully set aside in our study. The committees themselves remained dominated by males which, as has been reported elsewhere [37], can limit the full realization of empowerment. The second phase of evaluation also revealed that the operational changes necessitated by programme scale-up had an impact on external power relations: ‘ownership & leadership’ was perceived as being limited by the external partner which in this phase had assumed greater power related to the maintenance and sustainability of the project. This is one of the difficulties reported by many authors when trying to incorporate empowerment approaches within top-down programme [38, 39]. Most of the studies found, that aimed to evaluate of a community participation, reported on this imbalanced power between the community and the external partner, because of programme externally designed and imposed to communities [30, 36, 37, 40].

In addition to external and internal power structures, our study further revealed that stakeholder attitudes towards power relation dynamics can be barriers towards the realisation of (greater) empowerment. Committee members expressed a desire to continue to work with external partners even though their experience of power within such partnerships was unbalanced. This acceptance possibly suggests that the committees may not have been able to envisage a different type of partnership. As some authors have advocated previously, poor and marginalized people often lack a sense of control over their health and well-being, leading to a sense of fatalism, and a tendency to wait for outside actors and agencies to take control of local health problems [41]. Empowerment does not mean the involvement of an external partner has to end. The attitude and nature of the power relations between the community and the external professional partner(s) are important. Partnerships with power relations based on equity can be seen as social capital that could increase empowerment and even make actions stronger and more effective [42].

Implications from our study for tsetse control programmes are potentially threefold. Firstly, a Tiny Target programme as operationalised in DRC can result in community empowerment. If even greater levels of empowerment are sought then a revised programme structure and attitudes more supportive of empowerment objectives may be needed. The top-down nature of tsetse control programmes limits empowerment [43–45]. If community empowerment is an objective of a progamme then the top-down structure needs to change to a more holistic and intersectoral approach involving various stakeholders. These could be the communities themselves, NGOs or government departments involved with the environment, agriculture and other human health concerns (e.g., malaria, other NTDs). Bardosh has argued that one of the problems commonly faced by community-based projects concerned with controlling NTDs is that their vision is limited to the disease only because of their vertical nature [46]. More holistic and intersectoral approaches would benefit communities as they create more opportunities for experiencing partnerships and for prioritizing needs identified by communities. In the case of g-HAT in DRC, many professionals are now advocating for a switch from a vertical programme led by the PNLTHA to a horizontal programme as part of the local health structures [10].

Attitudes toward community participation require a change, and this is probably even more essential than changing structures. A shift needs to occur to ensure that empowerment is envisioned as an aim in its own right [19, 46–51]. Attitudes conducive to sharing power such as respecting community opinions, respecting culture, accommodating the community, being transparent and encouraging dialogue should be prioritized. For empowerment to progress, the attitudes of all stakeholders need to change, including the communities. However, health professionals hold more power initially and are best placed to encourage and foster positive attitudes toward community participation. Changing the attitudes of health professionals towards sharing of power may also help them to better understand community heterogeneity [52] and internal power dynamics. As suggested previously, participation is most likely to empower marginalized communities to exercise control of their lives through the transfer of power from health professionals to communities and through effective knowledge sharing [53, 54].

This study aimed to evaluate community participation in a participatory manner with committee members being the evaluators. Previously, this approach has been used only once for tsetse control in Africa [30]. The use of Rifkin’s framework has also not been widely used. We found only four references in the African context [30, 36, 37, 40] and only one of them was participatory with indicators and the scoring evaluation made by community members [30]. Most previous studies that have used the Rifkin framework have applied the five pre-determined indicators used in the original version: leadership, organisation, need assessment, resource mobilisation and management [32]. Consequently, comparisons between our evaluation and these earlier studies are difficult. Some of the indicators that the committee members in our study chose to define community participation are comparable [52, 55, 56]. For example, the indicator ‘autonomy’ in our study relates to ‘resource mobilisation’ as it pertains to how community members contribute with their local resources. Other indicators have very different meanings even if using the same name. ‘Organisation’ in the original framework referred to whether the project was conceived with existing community structures, unlike our study where ‘organisation’ referred to who is responsible for implementing an action. The ‘willingness’ indicator in our study is also seemingly unique, with no obvious counterpart in previously published studies of community participation. This shows that definitions of community participation differ according to various and changing contexts. Accordingly, we would recommend further use of the adapted version of the Rifkin framework, by Draper et al. [33], that allows communities to define their own indicators of community participation.

## Limitations

Our study has several limitations. First, the scores given during the community participation analysis may have been subject to social desirability bias. Committee members had formed good relations with the researchers and were perhaps reluctant to disappoint; they may have been wary of upsetting external partners and/or losing support from the Tiny Targets project. Second, critical self-reflection is not necessarily a common practice for committee members. Third, the study may also have been subject to some form of courtesy bias in which participants expressed that they wanted the Research Team to continue to work in their villages. Fourth, the analyses were limited to understanding community participation through the experience of a relatively small group of participants and this may has created a bias. As some authors have noted previously, the structure of community leadership is often historically or culturally determined to exclude marginalised groups, including women, young people and marginalised men [57]. As a result, a committee scoring their empowerment as being high does not mean that all committee interests have been taken into account. Broader community participation might have revealed new or conflicting perspectives. Finally, the three committees lacked gender balance meaning the study findings have a male-dominated perspective.

## Conclusion

The Tiny Target project was a source of empowerment for the vector control committee members. They expressed a desire to continue participating with the project and partners. However, they also identified that power structures and attitudes inside the committee and between the community and partners limited the empowerment process. If empowerment is to be an important objective of projects and programmes two important changes are suggested. First, the top down structure currently in place within the g-HAT programme is not the most appropriate for empowerment and a more holistic and intersectoral approach is recommended. Second attitude of sharing power need to be encouraged.

## References

[1] World Health Organisation. Trypanosomiase humaine africaine (maladie du sommeil) 2021. Available from: https://www.who.int/fr/news-room/fact-sheets/detail/trypanosomiasis-human-african-(sleeping-sickness).

[2] Büscher P, Cecchi G, Jamonneau V, Priotto G. Human African trypanosomiasis. Lancet. 2017;390(10110):2397–409. Epub 20170630. doi: 10.1016/S0140-6736(17)31510-6 . 28673422

[3] Tirados I, Esterhuizen J, Kovacic V, Mangwiro TN, Vale GA, Hastings I, et al. Tsetse Control and Gambian Sleeping Sickness; Implications for Control Strategy. PLoS Negl Trop Dis. 2015;9(8):e0003822. Epub 2015/08/13. doi: 10.1371/journal.pntd.0003822 ; PubMed Central PMCID: PMC4580652. 26267814 PMC4580652

[4] World Health Organisation. Global Health Observatory data repository, Number of new reported cases (T.b. gambiense) Data by country. Available from: http://apps.who.int/gho/data/node.main.A1636?lang=en.

[5] Willyard C. Putting sleeping sickness to bed. Nat Med. 2011;17(1):14–7. doi: 10.1038/nm0111-14 . 21217663

[6] Simarro PP, Cecchi G, Franco JR, Paone M, Diarra A, Priotto G, et al. Monitoring the Progress towards the Elimination of Gambiense Human African Trypanosomiasis. PLoS Negl Trop Dis. 2015;9(6):e0003785. Epub 2015/06/10. doi: 10.1371/journal.pntd.0003785 ; PubMed Central PMCID: PMC4461311. 26056823 PMC4461311

[7] Holmes P. First WHO Meeting of Stakeholders on Elimination of Gambiense Human African Trypanosomiasis. PLoS Negl Trop Dis. 2014;8(1–2). doi: 10.1371/journal.pntd.0003244 25340404 PMC4207655

[8] Jamonneau V, Ilboudo H, Kabore J, Kaba D, Koffi M, Solano P, et al. Untreated human infections by Trypanosoma brucei gambiense are not 100% fatal. PLoS Negl Trop Dis. 2012;6(6):e1691. Epub 2012/06/22. doi: 10.1371/journal.pntd.0001691 ; PubMed Central PMCID: PMC3373650. 22720107 PMC3373650

[9] Bucheton B, MacLeod A, Jamonneau V. Human host determinants influencing the outcome of Trypanosoma brucei gambiense infections. Parasite Immunol. 2011;33(8):438–47. Epub 2011/03/10. doi: 10.1111/j.1365-3024.2011.01287.x ; PubMed Central PMCID: PMC3427891. 21385185 PMC3427891

[10] Mulenga P, Chenge F, Boelaert M, Mukalay A, Lutumba P, Lumbala C, et al. Integration of Human African Trypanosomiasis Control Activities into Primary Healthcare Services: A Scoping Review. Am J Trop Med Hyg. 2019;101(5):1114–25. Epub 2019/09/05. doi: 10.4269/ajtmh.19-0232 ; PubMed Central PMCID: PMC6838596. 31482788 PMC6838596

[11] Mpanya A, Hendrickx D, Vuna M, Kanyinda A, Lumbala C, Tshilombo V, et al. Should I get screened for sleeping sickness? A qualitative study in Kasai province, Democratic Republic of Congo. PLoS Negl Trop Dis. 2012;6(1):e1467. Epub 2012/01/25. doi: 10.1371/journal.pntd.0001467 ; PubMed Central PMCID: PMC3260312. 22272367 PMC3260312

[12] Solano P, Torr SJ, Lehane MJ. Is vector control needed to eliminate gambiense human African trypanosomiasis? Front Cell Infect Microbiol. 2013;3:33. Epub 2013/08/06. doi: 10.3389/fcimb.2013.00033 ; PubMed Central PMCID: PMC3728477. 23914350 PMC3728477

[13] Shaw AP, Tirados I, Mangwiro CT, Esterhuizen J, Lehane MJ, Torr SJ, et al. Costs of using "tiny targets" to control Glossina fuscipes fuscipes, a vector of gambiense sleeping sickness in Arua District of Uganda. PLoS Negl Trop Dis. 2015;9(3):e0003624. Epub 20150326. doi: 10.1371/journal.pntd.0003624 ; PubMed Central PMCID: PMC4374750. 25811956 PMC4374750

[14] Shaw AP, Torr SJ, Waiswa C, Cecchi G, Wint GR, Mattioli RC, et al. Estimating the costs of tsetse control options: an example for Uganda. Prev Vet Med. 2013;110(3–4):290–303. Epub 20130228. doi: 10.1016/j.prevetmed.2012.12.014 . 23453892

[15] Esterhuizen J, Rayaisse JB, Tirados I, Mpiana S, Solano P, Vale GA, et al. Improving the cost-effectiveness of visual devices for the control of riverine tsetse flies, the major vectors of human African trypanosomiasis. PLoS Negl Trop Dis. 2011;5(8):e1257. Epub 2011/08/11. doi: 10.1371/journal.pntd.0001257 ; PubMed Central PMCID: PMC3149014. 21829743 PMC3149014

[16] Torr SJ, Hargrove JW, Vale GA. Towards a rational policy for dealing with tsetse. Trends Parasitol. 2005;21(11):537–41. Epub 20050902. doi: 10.1016/j.pt.2005.08.021 . 16140579

[17] Vander Kelen C, Mpanya A, Boelaert M, Pérez D, Pulford J, Selby R, et al. Feasibility of community-based control of tsetse: a pilot project using Tiny Targets in the Democratic Republic of Congo. PLoS Negl Trop Dis. 2020;14(9). 10.1371/journal.pntd.0008696 PMC7537905 32970689

[18] Rifkin SB. [The Alma-Ata declaration (OMS-UNICEF conference) (author’s transl)]. Rev Med Chil. 1979;107(5):452–3. 42130. 42130

[19] Madon S, Malecela MN, Mashoto K, Donohue R, Mubyazi G, Michael E. The role of community participation for sustainable integrated neglected tropical diseases and water, sanitation and hygiene intervention programs: A pilot project in Tanzania. Soc Sci Med. 2018;202:28–37. Epub 20180222. doi: 10.1016/j.socscimed.2018.02.016 ; PubMed Central PMCID: PMC5906643. 29501716 PMC5906643

[20] Rifkin SB. Alma Ata after 40 years: Primary Health Care and Health for All-from consensus to complexity. BMJ Glob Health. 2018;3(Suppl 3):e001188. Epub 20181220. doi: 10.1136/bmjgh-2018-001188 ; PubMed Central PMCID: PMC6307566. 30622747 PMC6307566

[21] Gouteux JP, Sinda D. Community participation in the control of tsetse flies. Large scale trials using the pyramid trap in the Congo. Trop Med Parasitol. 1990;41(1):49–55. Epub 1990/03/01. . 2339247

[22] Gouteux JP, Bansimba P, Bissadidi N, Noireau F. [Responsibility for tsetse control by rural communities: first trial in 5 Congolese villages]. Ann Soc Belg Med Trop. 1987;67(1):37–49. Epub 1987/03/01. . 3632076

[23] Laveissiere C, Meda HH. The campaign against sleeping sickness by trapping: not as easy as one thinks! Ann Soc Belg Med Trop. 1992;72 Suppl 1:57–68. Epub 1992/01/01. . 1329680

[24] Laveissiere C, Hervouet J, Couret D, Eouzan J, Mérouze F. La campagne pilote de lutte contre la trypanosomiase humaine dans le foyer de Vavoua (Côte d’Ivoire). Cah ORSTOM, sér, ENtméd et Parasitol. 1985;XXIII:167–85.

[25] Williams B, Campbell C, Willimas R. Broken House: Science and development in Africa. Preventive Veterinary Medicine. 1995;49:95–113.

[26] Lumbala C, Simarro PP, Cecchi G, Paone M, Franco JR, Kande Betu Ku Mesu V, et al. Human African trypanosomiasis in the Democratic Republic of the Congo: disease distribution and risk. Int J Health Geogr. 2015;14:20. Epub 20150606. doi: 10.1186/s12942-015-0013-9 ; PubMed Central PMCID: PMC4501122. 26047813 PMC4501122

[27] Franco JR, Cecchi G, Paone M, Diarra A, Grout L, Kadima Ebeja A, et al. The elimination of human African trypanosomiasis: Achievements in relation to WHO road map targets for 2020. PLoS Negl Trop Dis. 2022;16(1):e0010047. Epub 20220118. doi: 10.1371/journal.pntd.0010047 ; PubMed Central PMCID: PMC8765662. 35041668 PMC8765662

[28] Barrett K, Okali C. Community participation in the management of tsetse. A comparative assessment of impact and sustainability. DFID, 1998.

[29] Barrett K, Okali C. Partnership for tsetse control-community participation and other option. FAO, 1998.

[30] Kovacic V. Women-led tsetse control: A pilot Study in Northwest Uganda.: University of Liverpool; 2015.

[31] Rifkin SB. Paradigms lost: toward a new understanding of community participation in health programmes. Acta Trop. 1996;61(2):79–92. doi: 10.1016/0001-706x(95)00105-n . 8740887

[32] Rifkin SB, Muller F, Bichmann W. Primary health care: on measuring participation. Soc Sci Med. 1988;26(9):931–40. Epub 1988/01/01. doi: 10.1016/0277-9536(88)90413-3 . 3388072

[33] Draper AK, Hewitt G, Rifkin SB. Chasing the dragon: Developing indicators for the assessment of community participation in health programmes. Soc Sci Med. 2010;71(6):1102–9. Epub 20100619. doi: 10.1016/j.socscimed.2010.05.016 . 20621405

[34] Morgan LM. Community participation in health: perpetual allure, persistent challenge. Health Policy Plan. 2001;16(3):221–30. Epub 2001/08/31. doi: 10.1093/heapol/16.3.221 . 11527862

[35] Green J, Thorogood N. Qualitative Mthods for Health research. 3rd ed. London: Sage; 2004 2014.

[36] Schmidt D, Rifkin SB. Measuring Participation: Its use as a managerial tool for district health planners based on a case study in Tanzania. International Journal of Health Planning and Management. 1996;11:345–58. doi: 10.1002/(SICI)1099-1751(199610)11:4&lt;345::AID-HPM445&gt;3.0.CO;2-F 10164455

[37] Baatiema L, Skovdal M, Rifkin S, Campbell C. Assessing participation in a community-based health planning and services programme in Ghana. BMC Health Serv Res. 2013;13:233. Epub 20130626. doi: 10.1186/1472-6963-13-233 ; PubMed Central PMCID: PMC3733901. 23803140 PMC3733901

[38] Kay BH, Tuyet Hanh TT, Le NH, Quy TM, Nam VS, Hang PV, et al. Sustainability and cost of a community-based strategy against Aedes aegypti in northern and central Vietnam. Am J Trop Med Hyg. 2010;82(5):822–30. doi: 10.4269/ajtmh.2010.09-0503 ; PubMed Central PMCID: PMC2861387. 20439962 PMC2861387

[39] Toledo ME, Vanlerberghe V, Baly A, Ceballos E, Valdes L, Searret M, et al. Towards active community participation in dengue vector control: results from action research in Santiago de Cuba, Cuba. Trans R Soc Trop Med Hyg. 2007;101(1):56–63. Epub 20060705. doi: 10.1016/j.trstmh.2006.03.006 . 16824565

[40] Barker M, Klopper H. Community participation in primary health care projects of the Muldersdrift Health and Development Programme. Curationis. 2007;30(2):36–47. doi: 10.4102/curationis.v30i2.1070 . 17703821

[41] Campbell CJ S. Health, community and development: towards a social psychology of participation. J community Appl Soc Psychol. 2000;10(4):255–70.

[42] Laverack. Adressing the contradiction between discourse and practice in health promotion. Deakin Univesity: Deakin University; 1999.

[43] Rifkin SB. Lessons from community participation in health programmes: a review of the post Alma-Ata experience. Int Health. 2009;1(1):31–6. doi: 10.1016/j.inhe.2009.02.001 . 24036293

[44] Wallerstein N. Powerlessness, empowerment, and health: implications for health promotion programs. Am J Health Promot. 1992;6(3):197–205. doi: 10.4278/0890-1171-6.3.197 . 10146784

[45] Atkinson JA, Vallely A, Fitzgerald L, Whittaker M, Tanner M. The architecture and effect of participation: a systematic review of community participation for communicable disease control and elimination. Implications for malaria elimination. Malar J. 2011;10:225. Epub 20110804. doi: 10.1186/1475-2875-10-225 ; PubMed Central PMCID: PMC3171376. 21816085 PMC3171376

[46] Bardosh K. Global aspirations, local realities: the role of social science research in controlling neglected tropical diseases. Infect Dis Poverty. 2014;3(1):35. Epub 20141001. doi: 10.1186/2049-9957-3-35 ; PubMed Central PMCID: PMC4197218. 25320672 PMC4197218

[47] Shediac-Rizkallah MC, Bone LR. Planning for the sustainability of community-based health programs: conceptual frameworks and future directions for research, practice and policy. Health Educ Res. 1998;13(1):87–108. doi: 10.1093/her/13.1.87 . 10178339

[48] Oino P, Towett G, Kirui KK, Luvega C. The Dilemma in Sustainability of Community-based Projects in Kenya. Global Journal of Advanced Research. 2015;2(4):757–68.

[49] Adekeye OA, Dean L, Dixon R. Community Engagement in Neglected Tropical DiseaseTreatment in Nigeria: Rethinking the needs of varying contexts. Countdown. 2017.

[50] Ackley C, Elsheikh M, Zaman S. Scoping review of Neglected Tropical Disease Interventions and Health Promotion: A framework for successful NTD interventions as evidenced by the literature. PLoS Negl Trop Dis. 2021;15(7):e0009278. Epub 20210706. doi: 10.1371/journal.pntd.0009278 ; PubMed Central PMCID: PMC8321407. 34228729 PMC8321407

[51] Manderson L, Aagaard-Hansen J, Allotey P, Gyapong M, Sommerfeld J. Social research on neglected diseases of poverty: continuing and emerging themes. PLoS Negl Trop Dis. 2009;3(2):e332. Epub 20090224. doi: 10.1371/journal.pntd.0000332 ; PubMed Central PMCID: PMC2643480. 19238216 PMC2643480

[52] Laverack G, Wallerstein N. Measuring community empowerment: a fresh look at organizational domains. Health Promot Int. 2001;16(2):179–85. doi: 10.1093/heapro/16.2.179 . 11356756

[53] Chambers R. Participatory Rural Appraisal (PRA): Analysis of experience. worl development. 1994;22(9):1253–68.

[54] Freire P. La educación liberadora. Mexico DF: Siglo XXI; 1981.

[55] De Vos P, Al. e. Comprehensive Participatory Planning and Evaluation (CPPE). Social Medicine. 2011;6(2):106–17.

[56] Perez D, Lefevre P, Romero MI, Sanchez L, De Vos P, Van der Stuyft P. Augmenting frameworks for appraising the practices of community-based health interventions. Health Policy Plan. 2009;24(5):335–41. Epub 20090623. doi: 10.1093/heapol/czp028 . 19549795

[57] Goodman RM, Speers MA, McLeroy K, Fawcett S, Kegler M, Parker E, et al. Identifying and defining the dimensions of community capacity to provide a basis for measurement. Health Educ Behav. 1998;25(3):258–78. doi: 10.1177/109019819802500303 . 9615238

